# Topological valley-locked waveguides with C_4_ impurity

**DOI:** 10.1515/nanoph-2024-0192

**Published:** 2024-06-19

**Authors:** Hongxiang Zhang, Rensheng Xie, Xiaofeng Tao, Jianjun Gao

**Affiliations:** Key Laboratory of Polar Materials and Devices, Department of Electronic Sciences, School of Physics and Electronic Sciences, 12655 East China Normal University, Shanghai 200241, China; Centre for Disruptive Photonic Technologies, School of Physical and Mathematical Sciences & The Photonics Institute, Nanyang Technological University, Singapore 639798, Singapore; National Engineering Research Centre of Mobile Network Technologies, Beijing University of Posts and Telecommunications, Beijing 100876, China

**Keywords:** heterostructure, valley-locked waveguides, C_4_ impurity, coding channels, energy concentrators

## Abstract

Heterostructures play a pivotal role in the design of valley-locked waveguides, facilitating the manipulation of width as an additional degree of freedom. Through this design, we demonstrate the extension of the topological guided modes from the domain wall of topologically nontrivial valley photonic crystals (VPCs) into the trivial VPCs. We propose a C_4_ impurity to control the states of the light wave transmission in topological valley-locked waveguides through the intervalley scattering of defects in Quantum Valley Spin Hall topological insulators. By rotating the C_4_ structure, the ON/OFF (0°/45°) state of the valley-locked waveguides can be controlled, effectively serving as a switch component. Furthermore, many unique applications could be devised based on the introduced impurity. Examples include the development of coding channels with arbitrary output ports and energy concentrators with enhanced secondary concentration. The proposed topological valley-locked waveguides with C_4_ impurity will be beneficial for on-chip integrated photonic networks.

## 1 Introduction

Topological valley edge states, recognized for their gapless dispersion and capacity to induce unidirectional transport, have undergone extensive investigation in various fields including electronics [1], [2], [3], [4], photonics [5], [6], [7], [8], and acoustics [9], [10], [11], [12] in recent years. Utilizing the concept of bulk-edge correspondence, the valley edge states emerge through the use of valley photonic crystals characterized by opposite valley Chern numbers [13], [14], [15]. The valley edge states appear at the interface of the domains with opposite valley Chern numbers or band inversion, which are gapless and topological valley-locked [16], [17]. Recently, on-chip photonic devices have generated significant attention due to their advantages, such as low energy consumption and wide bandwidth, in information processing [18], [19]. However, the inevitable imperfections in the fabrication of photonic devices diminish their performance [20]. The advent of topological photonics has introduced a solution to mitigate the functional impact of fabrication errors in photonic devices [21], [22]. It has evolved into a pivotal platform in modern photonic devices, encompassing topological channel intersections [23], [24], topological lasers [25], [26], and robust on-chip communications [27], [28], [29], [30].

In contrast to the guided modes in conventional waveguides, such as silicon rib waveguides and rectangular waveguides [31], [32], valley edge states typically localize at the interface of two VPCs with opposite valley Chern numbers. This confinement results in the width of topological waveguides being untunable. The tunability of waveguide width provides a degree of freedom (DOF) for manipulating guided modes and designing photonic devices. Notably, the waveguides with width DOF exhibit high flexibility in interfacing with other photonic devices [33], [34], [35]. To address the above challenges, researchers have focused on the width DOF of topological waveguides. Over the past years, width-tunable topological waveguides have been investigated in both optics and acoustics, employing specific heterostructures [9], [11], [17],[36], [37], [38]. These waveguides possess a distinctive capability to adjust their width, offering control and versatility in guiding light or sound waves while leveraging the principles of topological physics [39], [40], [41], [42].

In this paper, we introduce width DOF into VPCs waveguides to enhance the flexibility in manipulating the propagation of light waves. Specifically, we construct topological valley-locked waveguides (TVLWs) using a heterostructure, where a VPC characterized by Dirac cones is sandwiched between two VPCs with opposite valley Chern numbers [9], [11], [43]. The heterostructures enable the extension of topological guided modes (TGMs) from the domain wall of topologically nontrivial VPCs into the trivial VPCs, featuring a closed bandgap. This extension introduces a width DOF for wave transmission. The proposed TVLWs support defect-immune edge state transmission, exhibiting features such as gapless dispersion, TGMs, and tunable width. However, the TVLWs are not robust to all types of defects. For instance, in Quantum Spin Hall (QSH) topological insulators, defects causing spin-mixing can lead to backscattering [44], [45], and certain defects and corners introduced into intervalley scattering in Quantum Valley Spin Hall (QVSH) topological insulators [21], [46], [47], [48]. The strategic utilization of defects can help design photonic devices to manipulate light transmission. Thus, we design a C_4_ impurity composed of four identical metallic blades around the mid-metallic rod. By rotating the C_4_ structure, the ON/OFF (0°/45°) state of the TVLWs can be controlled, effectively serving as a switch component. The calculated switch ratio can even reach 63.5 dB [49], [50]. Moreover, leveraging the states of impurity, we create a coding channel with varying switch states and input ports to achieve arbitrary output signals. Notably, TVLWs exhibit resilience to sharp corners and bends, allowing for a significant reduction in waveguide width to achieve energy concentrations. This designed concentrator not only enhances field strength but also enables intense light–matter interactions, presenting a novel approach to flexibly control light transmission. In addition, with the assistance of the impurity in the “OFF” state, the concentrator can undergo secondary concentration. Devices utilizing heterostructures showcase immense potential across various applications and are expected to overcome the challenges in micro–nano fabrication, leading to a more efficient and effective fabrication process and information processing.

## 2 Results and discussion

### 2.1 Design of extended valley-locked structures

**Figure 1:**
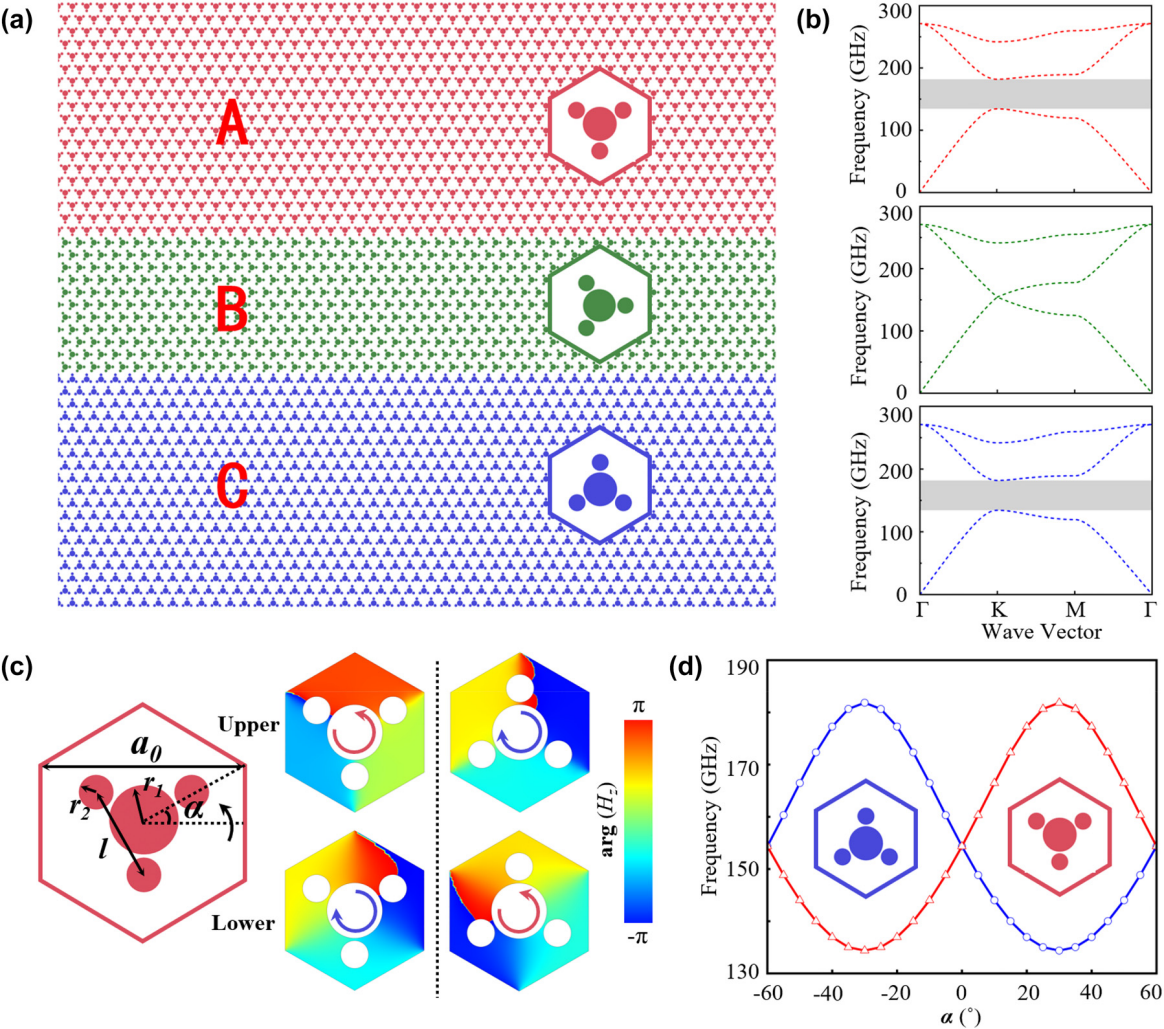
Heterostructure $\text{A}\left|{\text{B}}_{x}\right|\text{C}$ , with *x* being the number of layers in domain B. (a) Schematic of $\text{A}\left|{\text{B}}_{x}\right|\text{C}$ structures, red, green, and blue represent the unit A, B, and C, respectively. (b) The dispersion diagrams of unit A, B, and C, corresponding to the colors. The frequency of Dirac point in unit B is 154.35 GHz. The gray regions in unit A and C represent opening band gaps (134.38–181.58 GHz). (c) The detailed parameters of the unit cell, the lattice constant *a*_0_ = 500 μm, the radii of middle and concomitant metallic rods are *r*_1_ = 0.2*a*_0_ and *r*_2_ = 0.1*a*_0_, the distance between each concomitant rod is *l* = 0.55*a*_0_, and the rotation angle (*α*) is measured from the positive axis (30°, 0°, and −30° refer to unit A, B, and C, respectively). The phase distributions (arg(Hz)) of upper and lower band at the K valley in unit A and C. (d) The evolution of the photonic band gaps with the rotation angle *α*.

The design of TVLW consists of three domains $\text{A}\left|{\text{B}}_{x}\right|\text{C}$ (the heterostructure, see Figure 1(a)), where *x* denotes the number of layers in domain B. Each unit cell is constructed by four metallic rods embedded in a fused silica background (with a refractive index *n* = 2). More detailed parameters are shown in Figure 1(c), the lattice constant *a*_0_ = 500 μm, the radii of middle and concomitant metallic rods are *r*_1_ = 0.2*a*_0_ and *r*_2_ = 0.1*a*_0_, the distance between each concomitant rod is *l* = 0.55*a*_0_, and the rotation angle (*α*) is measured from the positive axis (30°, 0°, and −30° refer to units A, B, and C, respectively). Figure 1(b), calculated by a commercial software package (COMSOL Multiphysics) with Floquet boundary conditions, illustrates that the unit B features a Dirac degeneracy at the frequency of 154.35 GHz in K/K′ valley because of C_3v_-symmetry. With a rotation of the structure, the space-inversion symmetry in unit B will be broken from C_3v_ to C_3_, resulting in a bandgap (gray region) from 134.38 to 181.85 GHz. The phase patterns (arg(Hz)) of the upper and lower bands at K points for units A and C are depicted in Figure 1(c), revealing opposite winding directions in the phases of K valley states around the center of the units A and C at the same band. Additionally, the phases exhibit opposite directions within the same unit but different bands. The rotation angle *α* controls the Dirac mass terms and the corresponding valley-Chern number. Figure 1(d) describes the continuous evolution of a pair of frequency extreme points as the rotation angle *α* (from −60° to 60°) changes, elucidating the emergence of bandgap and the process of bands inversion.

**Figure 2:**
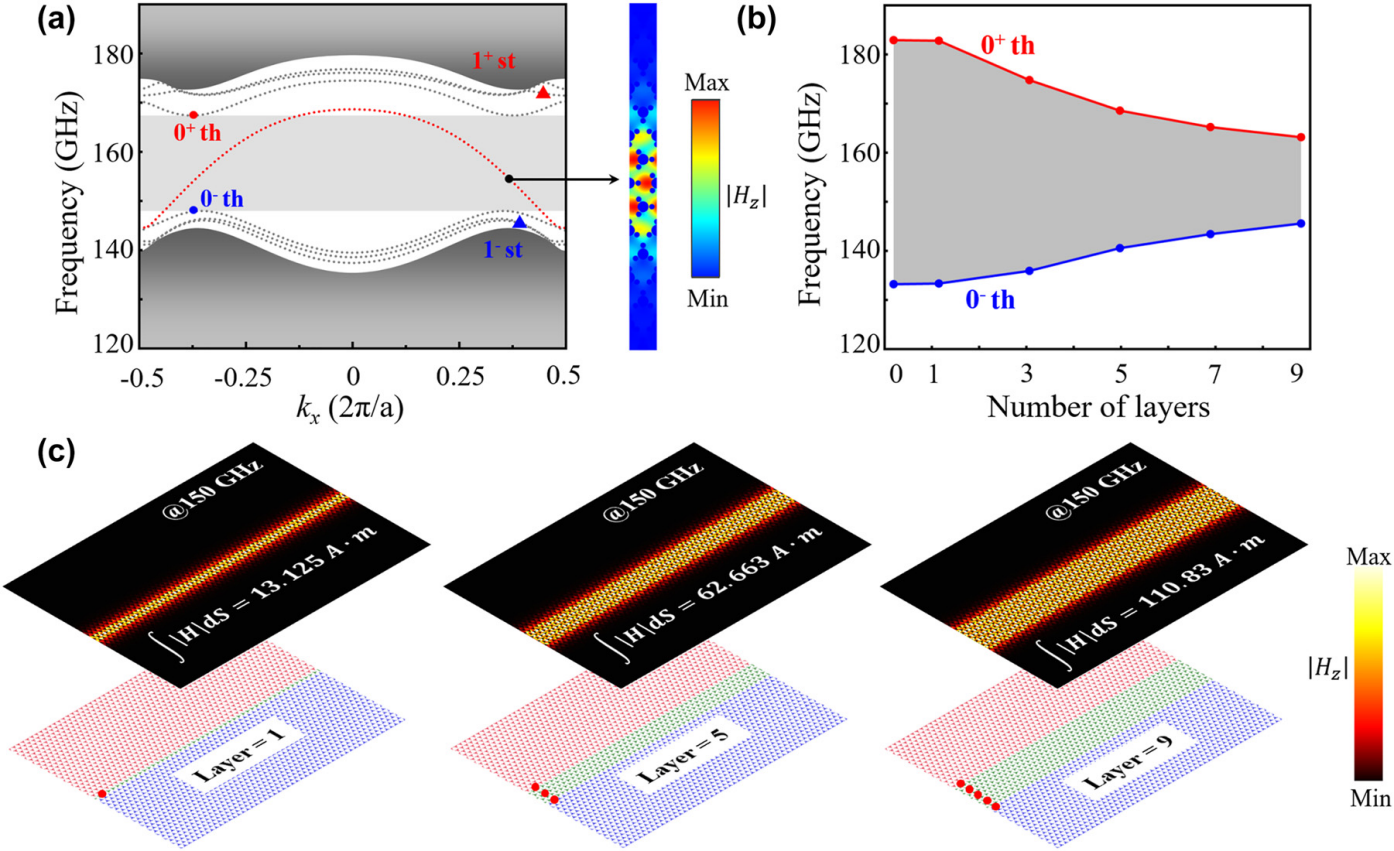
Topological edge states and guided mode distributions in $\text{A}\left|{\text{B}}_{x}\right|\text{C}$ . (a) Topological edge states of $\text{A}\left|{\text{B}}_{3}\right|\text{C}$ structures, the red dotted line represents the gapless TGM. The gray dotted lines represent the higher-order non-TGMs with gapped states, distinguished by the symbols of solid dots (red and blue represent 0^+^ th and 0^−^ th, respectively) and triangles (red and blue represent 1^+^ st and 1^−^ st, respectively). The light gray region depicts the topological frequency window. The right panel is simulated $\left|H_{z}\right|$ distribution of TGM. (b) The topological frequency window of different number *x*. (c) Lower panels: schematics of three TVLWs with *x* = 1, 5, and 9, red-marked points represent the sources. Upper panels: simulated $\left|H_{z}\right|$ distributions and the integration of |*H*| on the cross section of domain B at the frequency of 150 GHz.

Figure 2(a) depicts the dispersion diagram of the guided modes in the TVLW with layers *x* = 3 ( $\text{A}\left|{\text{B}}_{x}\right|\text{C}$ ). To calculate the edge states, we construct a supercell with 15 unit cells (6 unit A, 3 unit B, and 6 unit C, arranged from top to bottom) in the *y* direction and 1 period in the *x* direction. The Floquet period (Scattering) condition boundary is applied along the *x* (*y*) direction. The red dotted line represents the gapless TGM in the bulk bandgap. The group velocity of gapless TGM confined within the K/K′ valley displays an exact opposite behavior, demonstrating the valley-chirality locking property. This phenomenon bears a resemblance to the valley edge states in the $\text{A}\left|\text{C}\right.$ structure. The gray regions are the bulk bands. The simulated $\left|H_{z}\right|$ distribution of TGM is presented in the right panel of Figure 2(a). It can be seen that the energy concentrates the entire domain B and decays into A and C. Additionally, the gray dotted lines represent the higher-order non-TGMs with gapped states, distinguished by the symbols of solid dots (red and blue represent 0^+^ th and 0^−^ th, respectively) and triangles (red and blue represent 1^+^ st and 1^−^ st, respectively). Non-TGMs are clearly gapped and lack the valley-locking property. The light gray area between the 0^+^ th and 0^−^ th modes is referred as the topological frequency window, where only the TGM exists. As shown in Figure 2(b), the topological frequency window is determined by the layers of domain B, decreasing as *x* in domain B increases. At the same time, the other higher-order non-TGMs appear in the bulk. To comprehend this, one can envisage an extreme condition that, as *x* in domain B reaches a significant magnitude, the bulk-edge correspondence between domain A and C weakens considerably. Consequently, the topological frequency range gradually narrows, eventually leading to the reproduction of the Dirac points observed in the unit B.

The TGM in the TVLW can be intuitively elucidated through an effective Hamiltonian model. In accordance with the *k*⋅*p* theory, the effective Hamiltonian of the unit A, B, and C around the K valley can be written as

(1) $$\delta H_{K}\left(\delta k\right)=\upsilon_{D}\delta k_{x}\sigma_{x}+\upsilon_{D}\delta k_{y}\sigma_{y}+m\upsilon_{D}^{2}\sigma_{z}$$

where *δk* = *k* − *k*_*K*_ is the displacement of wave vector *k* to the K valley in the reciprocal space, *υ*_*D*_ is the group velocity (the slope of the linear Dirac cone in unit B), and *σ*_*i*_ (*i* = *x*, *y*, *z*) are elements in the Pauli matrices. *m* is the effective mass term, with *m* < 0 for unit A, *m* = 0 for unit B, and *m* > 0 for unit C. Applying the time-reversal operation allows us to derive the effective Hamiltonian around the K′ valley. The band inversion occurring between unit A and C corresponds directly to the reversal in the sign of the Dirac mass for the two photonic crystals. From the eigenvalue equation *δH*_*K*_ *ϕ* = *δωϕ*, the dispersion relation of unit A, B, and C can be derived as

(2) $$\delta^{2}\omega =\upsilon_{D}^{2}\left(\delta^{2}k_{x}+\delta^{2}k_{y}\right)+m^{2}\upsilon_{D}^{4}$$

In addition, if the TGM, *ϕ*_*ABC*_, exists, it is expected to exponentially attenuate along the +*y* direction in domain A and along −*y* direction in domain C. A specific solution featuring *δω* = *υ*_*D*_ *δk*_*x*_ and $\phi_{\mathrm{ABC}}={\left(1,1\right)}^{T}$ can be obtained, whose slope *υ*_*D*_ is the same as that of bulk states in unit B (*δω* = ±*υ*_*D*_ *δk*). The band inversion in unit A and C and Dirac cone dispersion in unit B collectively contribute to the existence of TGM. In other word, the TGM is a combination of the edge states in $\text{A}\left|\text{C}\right.$ domain and the bulk states in domain B.

Obviously, a waveguide with width DOF based on TGM is significant for efficient energy transport in subsequent device designs. To demonstrate this, simple cases have been conducted. As illustrated in Figure 2(c), the lower panel schematically shows three TVLWs with *x* = 1, 5, and 9, where red-marked point sources are placed (1, 3, and 5 identical point sources for *x* = 1, 5, and 9, respectively, guaranteeing the uniform energy distributions along the waveguide). The upper panel displays the corresponding $\left|H_{z}\right|$ field distributions at the frequency of 150 GHz. In the upper panel of field distributions, energy is well confined in the domain B for all three cases. Besides, the integration of |*H*| energy on the waveguide cross section (domain B area) has been calculated, which notably reveals the TVLWs with width DOF have a varying capacity for energy transport. The results of three TVLWs indicate that a TVLW with larger width (larger *x* in domain B) has a higher capacity for energy transport (i.e., 13.125 A m for *x* = 1, 62.663 A m for *x* = 5, 110.83 A m for *x* = 9, exponent of 10^−5^). Therefore, the TVLWs are more flexible than $\text{A}\left|\text{C}\right.$ structure in use of applications.

### 2.2 TVLWs with C_4_ impurity

**Figure 3:**
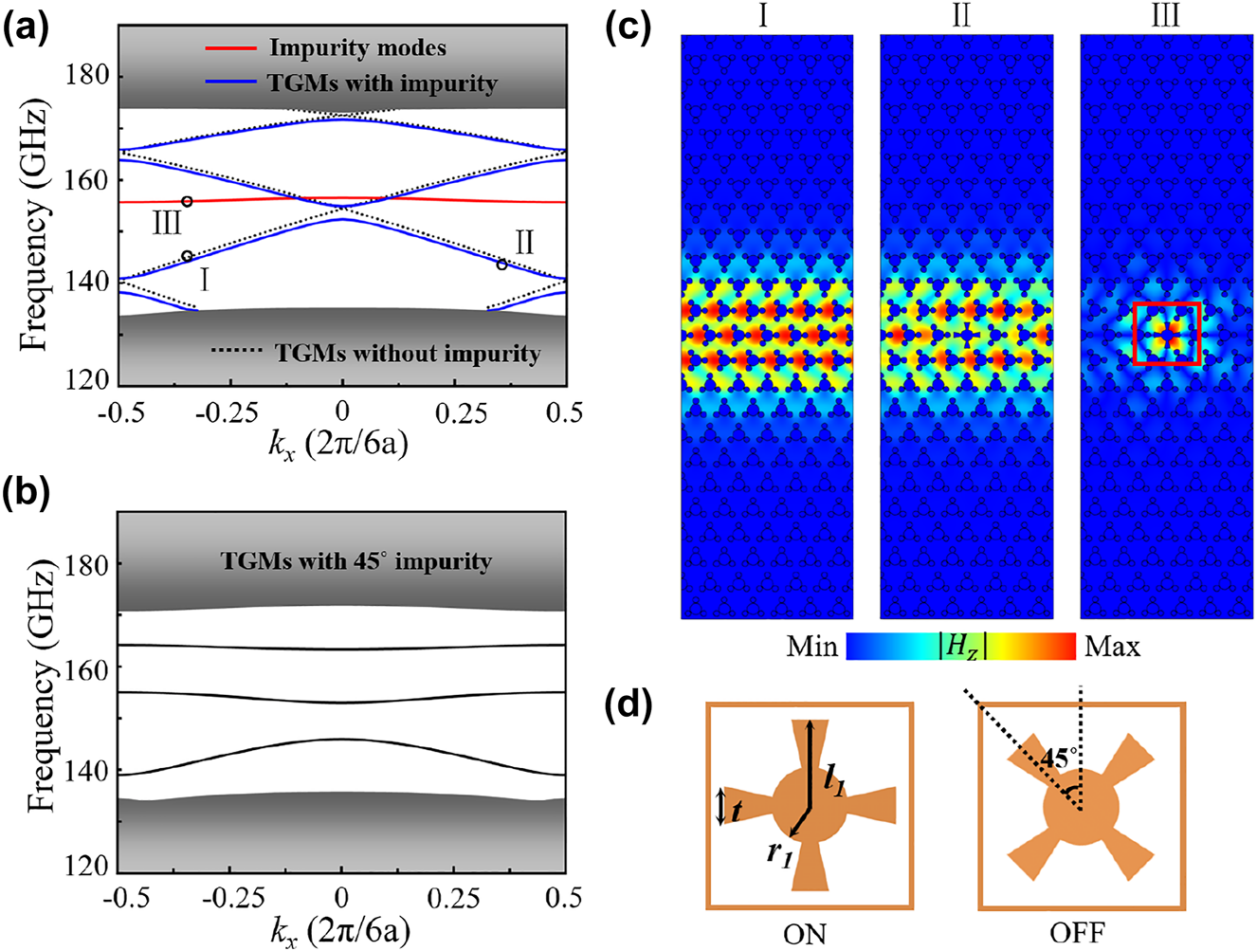
Band diagrams of TVLWs ( $\text{A}\left|{\text{B}}_{3}\right|\text{C}$ ) with C_4_ impurity. (a) The band diagrams of super-cell structure without impurity and with an “ON” impurity. (b) The band diagrams of super-cell structure with an “OFF” impurity. (c) Simulated $\left|H_{z}\right|$ distributions of the super-cell structure at points “I,” “II,” and “III” of (a). (d) The schematic of “ON” and “OFF” impurities. The lattice constant *a*_0_ = 500 μm, the radii of middle and concomitant metallic blades are *r*_1_ = 0.2*a*_0_, *l*_1_ = 0.45*a*_0_, and *t* = 0.2*a*_0_. The “OFF” impurity is rotated from the “ON” structure with a 45° angle.

The prominent advantages of TVLWs are robust against defects and intriguing splitting effect (see Supporting Information). Next, we focus on TVLWs with C_4_ impurity. The robustness of TVLWs is protected by C_3_ symmetry, according to ref. [21], a square structure can break the C_3_ symmetry. Thus, we further introduce a C_4_ impurity to study the robustness of TVLWs. As shown in Figure 3(d), in the fused silica background, the C_4_ impurity structure consists of four identical metallic blades (*l*_1_ = 0.45*a*_0_, *t* = 0.2*a*_0_), combined with a metallic rod (*r*_1_ = 0.2*a*_0_) in the middle. To demonstrate the effect of the C_4_ structure in the TVLWs, we design two cases of impurity (the left panel of Figure 3(d) defined as “ON” structure, the right panel of Figure 3(d) defined as “OFF” structure, by rotating the “ON” structure with a 45° angle). A six-period super-cell structure of $\text{A}\left|{\text{B}}_{3}\right|\text{C}$ (with *x* = 3) has been constructed, and the corresponding topological edge states are also calculated in the Figure 3(a) and (b).

In Figure 3(a), the black dotted lines represent the TGMs of the super-cell without an impurity, the solid lines represent the modes with an “ON” impurity (TGMs and impurity mode are distinguished by blue and red colors), and the gray regions mean the bulk edges (including the non-TGMs). We focus on the mid-guided modes for an example. Due to the zone-folding mechanism, guided modes (black dotted lines) with neighboring two branches degeneracy at high-symmetry points appear at the bulk gap [51]. When an “ON” impurity has been introduced into the super-cell structure, the initial degenerate points are split because of the breaking of the symmetry, and a band gap is formed between the two TGMs (blue solid lines). In addition, the impurity mode (red solid line) breaks the linear dispersion of the guided modes. The eigen field distributions at points “I,” “II,” and “III” are depicted in Figure 3(c), which exhibit the different guided mode characteristics. The energy is also confined in the domain B even with an “ON” impurity at the TGMs. When an “OFF” impurity is placed into the super-cell structure, the branches degeneracy points dramatically split (see Figure 3(b)). The upper bands become nearly flat, which lead to the slow light transmission (low group velocity). The existence of impurity can induce the intervalley scattering, which causes the reflection.

### 2.3 Applications of TVLWs with C_4_ impurity

**Figure 4:**
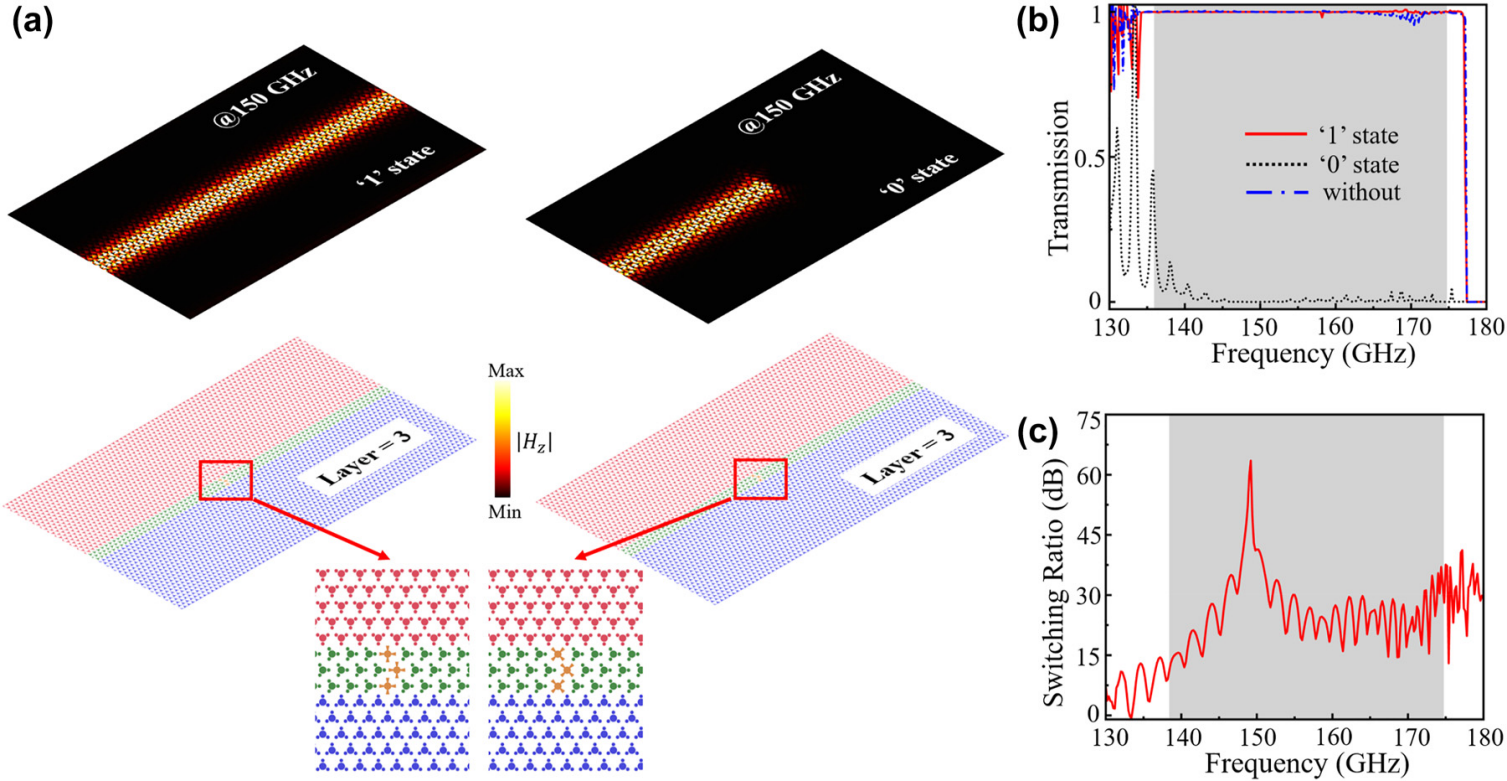
Switch on/off of TVLWs ( $\text{A}\left|{\text{B}}_{3}\right|\text{C}$ ) with C_4_ impurity. (a) The simulated $\left|H_{z}\right|$ distributions of switch on/off states. The enlarge part are the switch impurities. (b) The transmission spectrums of TVLWs at “1” state (red solid line), “0” state (black dotted line), and without impurities (blue dotted-solid line). (c) Calculated on/off switching ratios of TVLWs with C_4_ impurities.

Upon the properties of TVLWs with C_4_ impurity discussed above, here we design a switch to control wave propagation on/off based on $\text{A}\left|{\text{B}}_{3}\right|\text{C}$ . To better impede TVLWs transmission in the domain B, three “OFF” impurities are placed. Additionally, the TVLWs with “ON” (“OFF”) impurities are defined as “1” (“0”) state. As illustrated in Figure 4(a), two TVLWs with “ON” and “OFF” impurities (see mid panel of Figure 4(a)) have been designed to demonstrate the switch function. Figure 4(b) exhibits the transmission spectrum of TVLWs at “1” (red solid line) state, “0” (black dotted line) state and without impurities (blue dotted-solid line), with the gray region representing the topological frequency window. At “1” state, the transmission efficiency is near 1, similar to the normal TVLWs. While at “0” state, the spectrum are near zero from the 138.4–174.8 GHz. To further describe the switch function, in Figure 4(c), we calculate the on/off switching ratios for this switch, where the switching ratios can be defined as: $10\times \text{lo}{\text{g}}_{10}\left(T_{1}/T_{0}\right)$ , *T*_1_ and *T*_0_ are the transmission at “1” and “0” states, respectively. The gray area in Figure 4(c) represents the high switching ratios range, which is more than 10 dB in the range of topological frequency window, and the highest switching ratio is 63.5 dB at the frequency of 149.2 GHz. The upper panel of Figure 4(a) is the simulated $\left|H_{z}\right|$ distributions of “1” and “0” states of TVLWs at the frequency of 150 GHz. It is obvious that the energy confined in the domain B transports through the waveguide at the “1” state. However, at the “0” state, the energy encountering the “OFF” impurities will be obstructed and cannot propagate any further. The switch parts are enlarged in the lower panel of Figure 4(a). Subsequently, the topologically protected coding channels based on TVLWs with impurity switches have been realized. We first construct a forked splitter channel (see Supporting Information) as the on-chip transmission path based on $\text{A}\left|{\text{B}}_{3}\right|\text{C}$ TVLWs.

**Figure 5:**
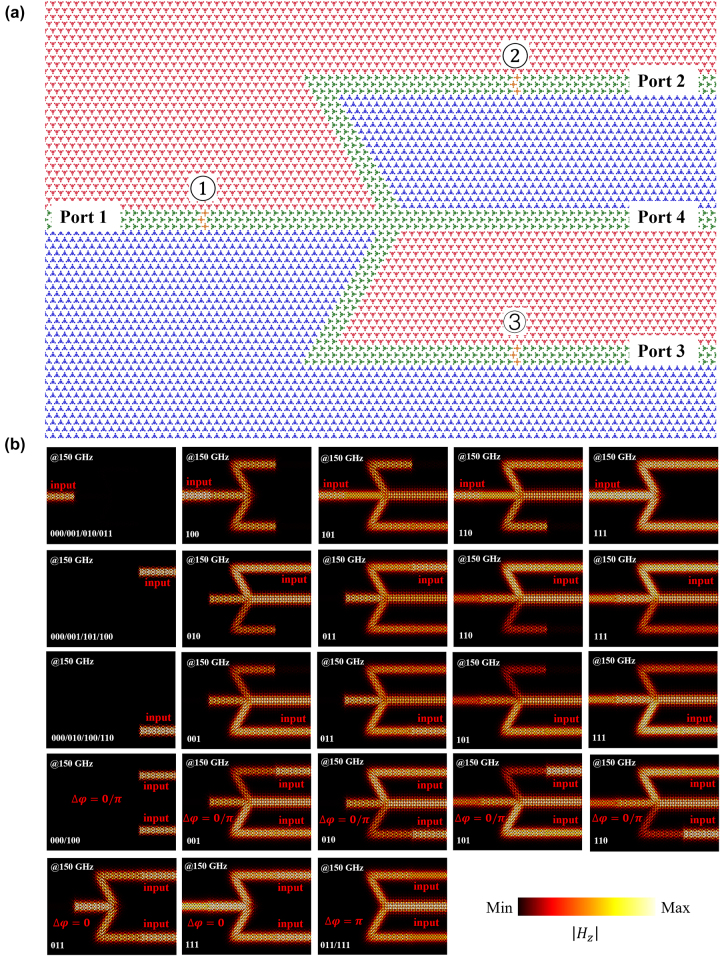
Schematic of coding channel based on TVLWs ( $\text{A}\left|{\text{B}}_{3}\right|\text{C}$ ). (a) The designed coding channel structure. (b) The simulated $\left|H_{z}\right|$ distributions of all the situations of input signals and switch states.

Then, combined with the previously designed switches, three switch components (the band diagrams of super-cell with 3 impurities see Supporting Information) are positioned on the left path (①), upper path (②), and the lower path (③), as depicted in Figure 5(a). By controlling the switch states and input ports, arbitrary output ports can be achieved. For example, when the channel is on “000” state (with all switches off), the input signal from any port cannot propagate to the other ports. Conversely, in the “111” state (with all switches on), equivalent to the channel without switches, the corresponding output signals are the same as the results verified above. However, switching off the ② (③), and inputting the signal from Port 1, the signals will outport from the Port 3 (Port 2) and Port 4. For the reason of switches placed at the ② (③) working as a reflector, it will produce a *π* phase difference. Furthermore, to control Port 4 more strictly, switches can also be placed on the path. Different from the results demonstrated above, the channel of Port 4 is $\text{C}\left|{\text{B}}_{3}\right|\text{A}$ , requiring the inversion states of switches (“0”/“1” state controls on/off of the $\text{C}\left|{\text{B}}_{3}\right|\text{A}$ TVLWs) for its inverse structure. All the situations of different input ports and different switch states without switching Port 4 are illustrated in Figure 5(b). We summarize all the kinds of switch states, input signals, and the corresponding output ports in Table 1.

**Table 1:** The different output signals with all input signals and switch states.

Input port	Switch states							
	000	001	010	011	100	101	110	111
1						3 & 4	2 & 4	2 & 3
2			4	3 & 4			1 & 4	1 & 3 & 4
3		4		2 & 4		1 & 4		1 & 2 & 4
2 & 3 (Δ*φ* = 0)		4	4			1 & 4	1 & 4	1
2 & 3 (Δ*φ* = *π*)		4	4			1 & 4	1 & 4	4

**Figure 6:**
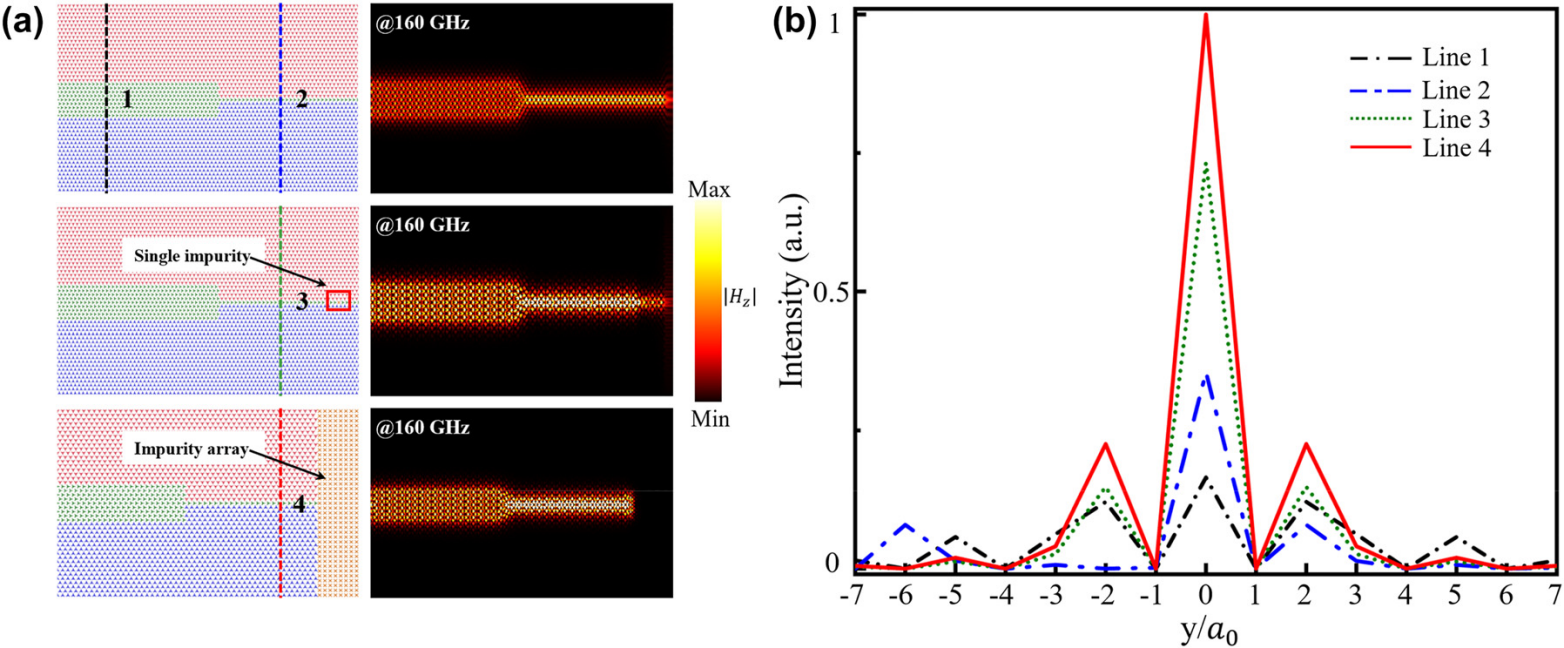
Energy concentrators based on TVLWs and impurities. (a) The schematic of energy concentrators with and without impurities. The left panel represents the actual structure, and the right panel represents the corresponding simulated $\left|H_{z}\right|$ distributions at the frequency of 160 GHz. (b) The normalized field intensity along line 1, line 2, line 3, and line 4 in (a).

Besides, achieving high-efficiency energy output remains a significant challenge in realizing an on-chip optical integration network characterized by a compact size and extensive integration. The number of layers *x* in domain B is independent because of the existence of TGMs. Leveraging the robustness and valley-locking properties of TVLWs, previously discussed, different numbers of layers in domain B can be combined to create a topological energy concentrator. For instance, a topological energy concentrator based on $\text{A}\left|{\text{B}}_{x}\right|\text{C}$ TVLWs is depicted in Figure 6(a) (see left panel), featuring an abrupt drop in the number of layers from 9 to 1. The simulated $\left|H_{z}\right|$ distribution at the frequency of 160 GHz is shown on the right panel in Figure 6(a), excited by a source at the left port of the concentrator. Exploiting the valley-locking property ensures forward propagation of only TGMs locked to the K valley, minimizing backward scattering. Furthermore, the robustness of TVLWs allows energy compression into narrower section of domain B. To assess energy transmission in domain B, the normalized intensity along the black and blue dotted lines, labeled Line 1 and Line 2 in Figure 6(a), is plotted in Figure 6(b). The field intensity of Line 2 is much larger than that of Line 1, indicating successful beam width compression and field enhancement. Additionally, when the switch, C_4_ impurity designed above, is at “OFF” state, it works as a reflector. Thus, we construct other two types of concentrators, with a single impurity and with an impurity array, as shown in the mid and lower panels in Figure 6(a), to achieve a secondary enhancement. The corresponding simulated $\left|H_{z}\right|$ distributions at the frequency of 160 GHz are plotted on the right panel in Figure 6(a), where the energy is more concentrated than that without impurity. We also calculate the field intensity at the similar position of the designed concentrators, marked by green and red dotted lines (labeled Line 3 and Line 4 in Figure 6(a)). All the intensity distributions are normalized in Figure 6(b), indicating that the energy will be secondarily concentrated with the impurities. This width-tunable energy concentrator with impurities offers a more compact and versatile alternative to traditional waveguides, promising potential applications in energy harvesting, taper-free waveguides, and other integrated photonic circuits.

## 3 Conclusions

In summary, we introduce a C_4_ impurity to control the states of TVLWs because of the intervalley scattering in QVSH topological insulators. Several applications based on TVLWs with impurity have been designed. Coding channels controlled by the states of switches and input signals, arbitrary output ports can be achieved. Utilizing the width DOF, an energy concentrator can be constructed. Moreover, concentrators with impurity proposed in this paper can be secondarily concentrated, contributing to energy harvesting and field enhancement. The TVLWs are more flexible to integrate with other photonic devices because of the width DOF, promoting the on-chip integrated networks and information processing.

## Supplementary Material

Supplementary Material Details
