# Supplementary material for: Topological valley-locked waveguides with C4 impurity
Source: Nanophotonics. 2024 Jun 19;13(19):3727–36. doi: 10.1515/nanoph-2024-0192 (PMC11465995; doi:10.1515/nanoph-2024-0192)
Supplement: Supplementary file 1 — Supplementary Material Details [file j_nanoph-2024-0192_suppl_001.docx]

**Supporting Information**

**Topological valley-locked waveguide with C_4_ impurity**

Hongxiang Zhang^1^, Rensheng Xie^3^, Xiaofeng Tao^2,*^, Jianjun Gao^1,*^

^1^Key Laboratory of Polar Materials and Devices, Department of Electronic Sciences, School of Physics and Electronic Sciences, East China Normal University, Shanghai 200241, China

^2^National Engineering Research Center of Mobile Network Technologies, Beijing University of Posts and Telecommunications, Beijing 100876, China

^3^Centre for Disruptive Photonic Technologies, School of Physical and Mathematical Sciences & The Photonics Institute, Nanyang Technological University, Singapore 639798, Singapore

*Corresponding Author

E-mail: [taoxf@bupt.edu.cn](mailto:taoxf@bupt.edu.cn), [jjgao@ee.ecnu.edu.cn](mailto:jjgao@ee.ecnu.edu.cn)

1. Properties of Topological valley-locked waveguides
2. The band diagrams of TVLWs with 3 impurities
3. Forked splitter channel with different input signals
4. Properties of Topological valley-locked waveguides

The prominent advantage of TVLWs lies in their robust transmission against defects. To verify this, four distinct kinds of defects (Bulging, Indentation, Bending, and Disorder) are introduced into domain B of the straight TVLWs ($A\left| B_{3} \right|C$, with $x=3$). As shown in Figs. S1(a)-(d), the upper panel depicts the $\left| H_{z} \right|$ distributions of the four structural defects at the frequency of 150 GHz, and the lower panel presents the schematics of TVLWs, respectively. A point source, similar to Fig. 2(c), is placed at the left of the TVLW to excite the TGM. Clearly, TVLWs possess the capability to propagate forward even in the presence of defects, owing to their inherent topological protection. In addition, we calculate the transmission of these four kinds straight TVLWs by integrating the energy flux at the input and output areas. In Fig. S1(e), the black, red, blue, and green lines represent the bulging, indentation, bending and disorder TVLWs, respectively. They all demonstrate high transmission efficiency in the topological frequency window (shadowed region). On the contrary, the $\left| H_{z} \right|$ distributions out of shadowed region show a large difference because of the higher-order non-TGMs. Moreover, we also simulate the same merits in $A\left| B_{5} \right|C$ (with $x=5$) to demonstrate the DOF of width, as depicted in Fig. S2.


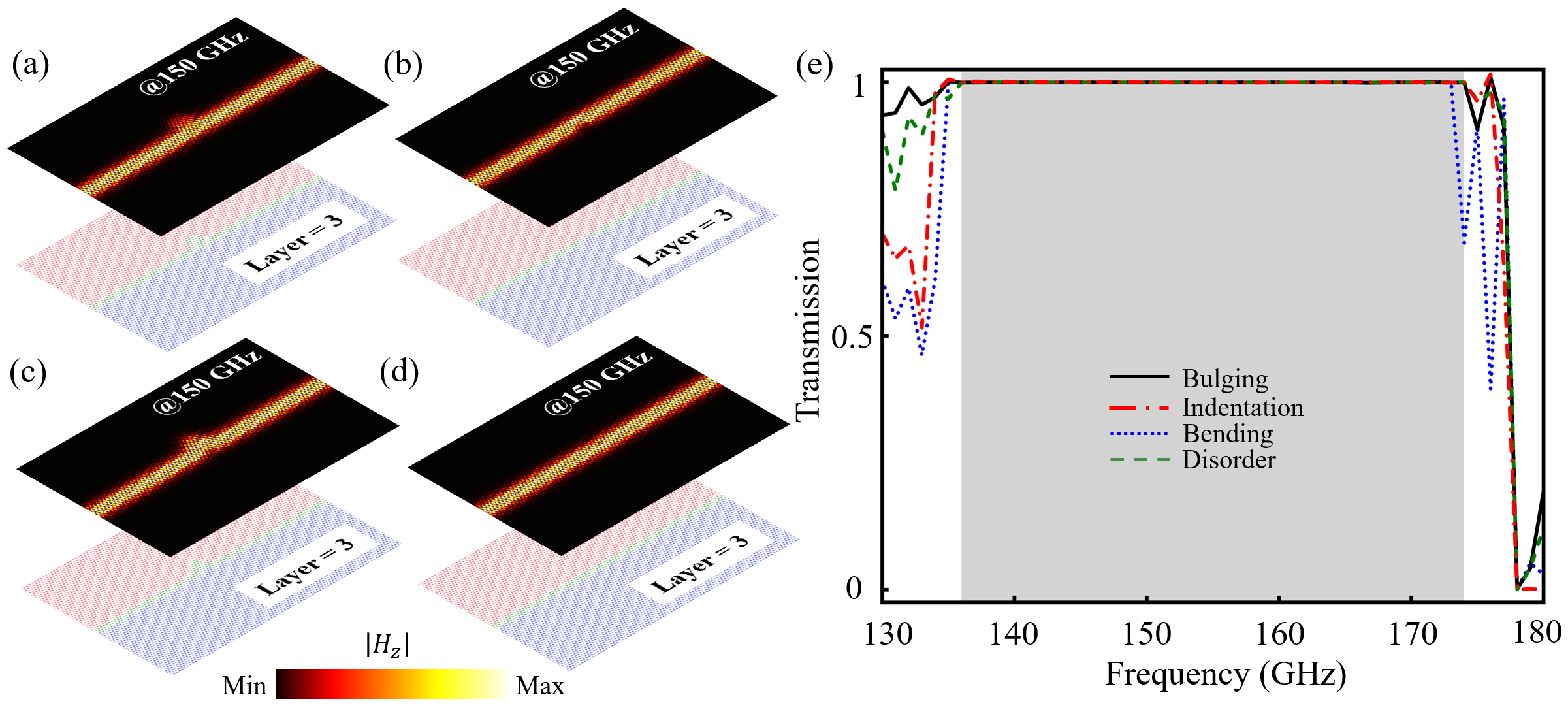


**Fig. S1.** Robustness of TVLWs against defects in $A\left| B_{3} \right|C$. Upper panels: simulated $\left| H_{z} \right|$ distributions at the frequency of 150 GHz. Lower panels: schematics of TVLWs with $x=3$. (a) Bulging, (b) Indentation, (c) Bending, (d) Disorder. (e) Transmission of four distinct kinds of defects in TVLWs. The black, red, blue, and green lines represent bulging, indentation, bending, and disorder, respectively. The topological frequency window is marked by grey region.


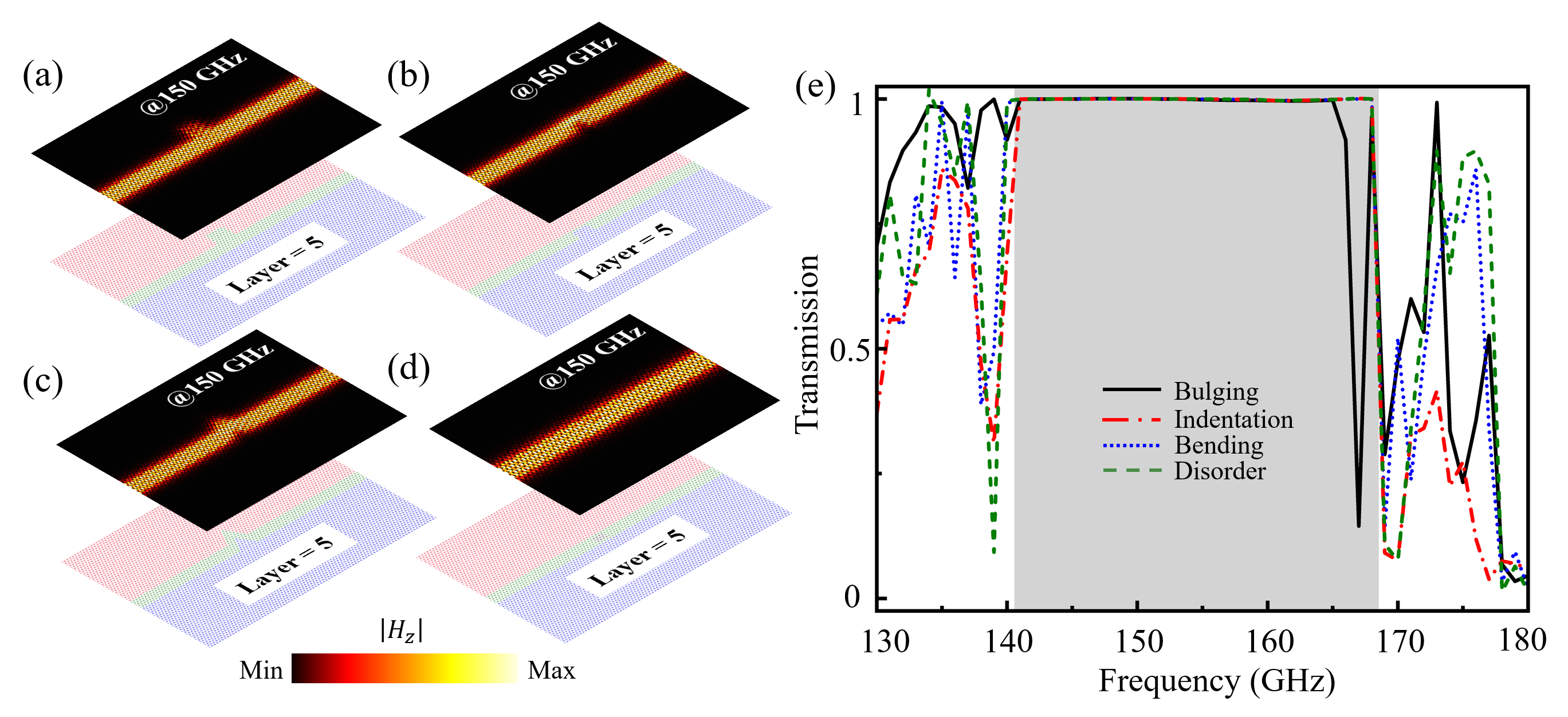


**Fig. S2.** Robustness of TVLWs against defects in $A\left| B_{5} \right|C$. Upper panels: simulated $\left| H_{z} \right|$ distributions at the frequency of 150 GHz. Lower panels: schematics of TVLWs with $x=5$. (a) Bulging, (b) Indentation, (c) Bending, (d) Disorder. (e) Transmission of four distinct kinds of defects in TVLWs. The black, red, blue, and green lines represent bulging, indentation, bending, and disorder, respectively. The topological frequency window is marked by grey region.

Valley-locked edge states are well-known for producing an intriguing splitting effect. To validate this phenomenon, we construct two types of valley-locked topological channel intersections based on TVLWs ($A\left| B_{3} \right|C$, with $x=3$), both consisting of five parts. Figs. S3(a) and (b) depict the two types of splitting channel structures, and we label four ports at the terminals of domain B as shown in the lower panel. Point sources are placed at Port 1 as input port to excite the TGM, while the Ports 2-4 are designated as output ports intended for signal reception. The $\left| H_{z} \right|$ distributions at the frequency of 150 GHz are shown in the upper panel of Figs. S3(a) and (b). Since the properties of TVLWs, the TGM only transports along the $A\left| B_{x} \right|C$ or $C\left| B_{x} \right|A$ waveguides, instead of $A\left| B_{x} \right|A$ and $C\left| B_{x} \right|C$. The field distributions illustrate this well, in Fig. S3(a), Ports 3 and 4 are suppressed, and the TGM can only transport to Port 2 channel. However, in Fig. S3(b), Port 2 is suppressed, and owing to the symmetry, the TGM is split into Ports 3 and 4, respectively. The calculated transmission efficiencies of these two types of intersections (see Figs. S3(c) and (d)) validate this phenomenon. The black, red, and blue lines represent the transmittances of three output ports, respectively. These simulation results indicate that TGM locked into the K/K’ valley propagate only along the corresponding types of TVLWs. Moreover, we also simulate the same merits in $A\left| B_{5} \right|C$ (with $x=5$) to demonstrate the DOF of width, as depicted in Fig. S4.


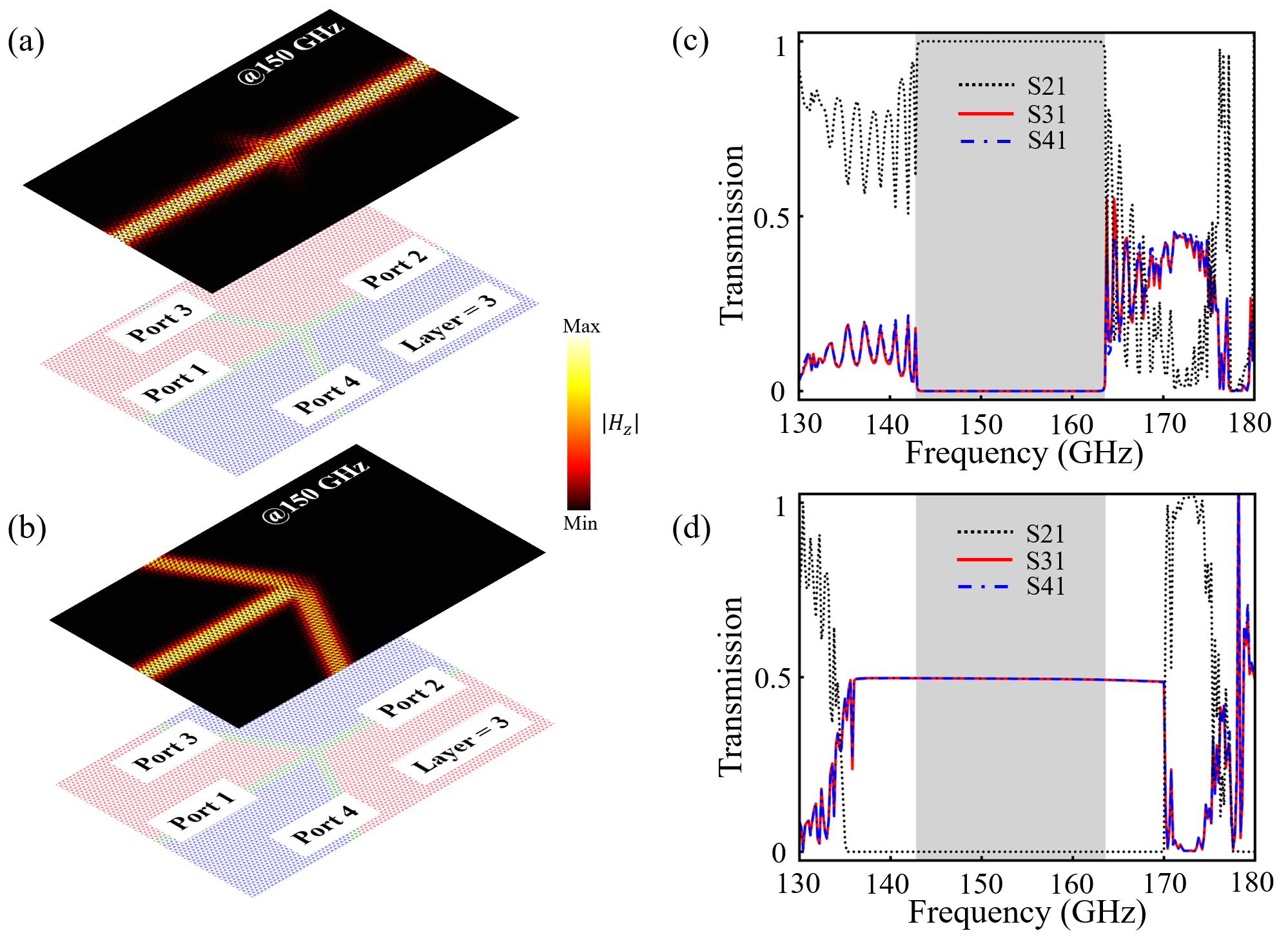


**Fig. S3.** Splitting effect in TVLWs. Upper panels: simulated $\left| H_{z} \right|$ distributions at the frequency of 150 GHz. Lower panels: schematics of TVLWs with $x=3$. (a) AACC, (b) ACCA. Transmission of two valley-locked topological channel. (c) AACC, (d) ACCA. The black, red, and blue lines represent transmission of ports 2-4, respectively. The grey region is the high-efficiency transmission range.


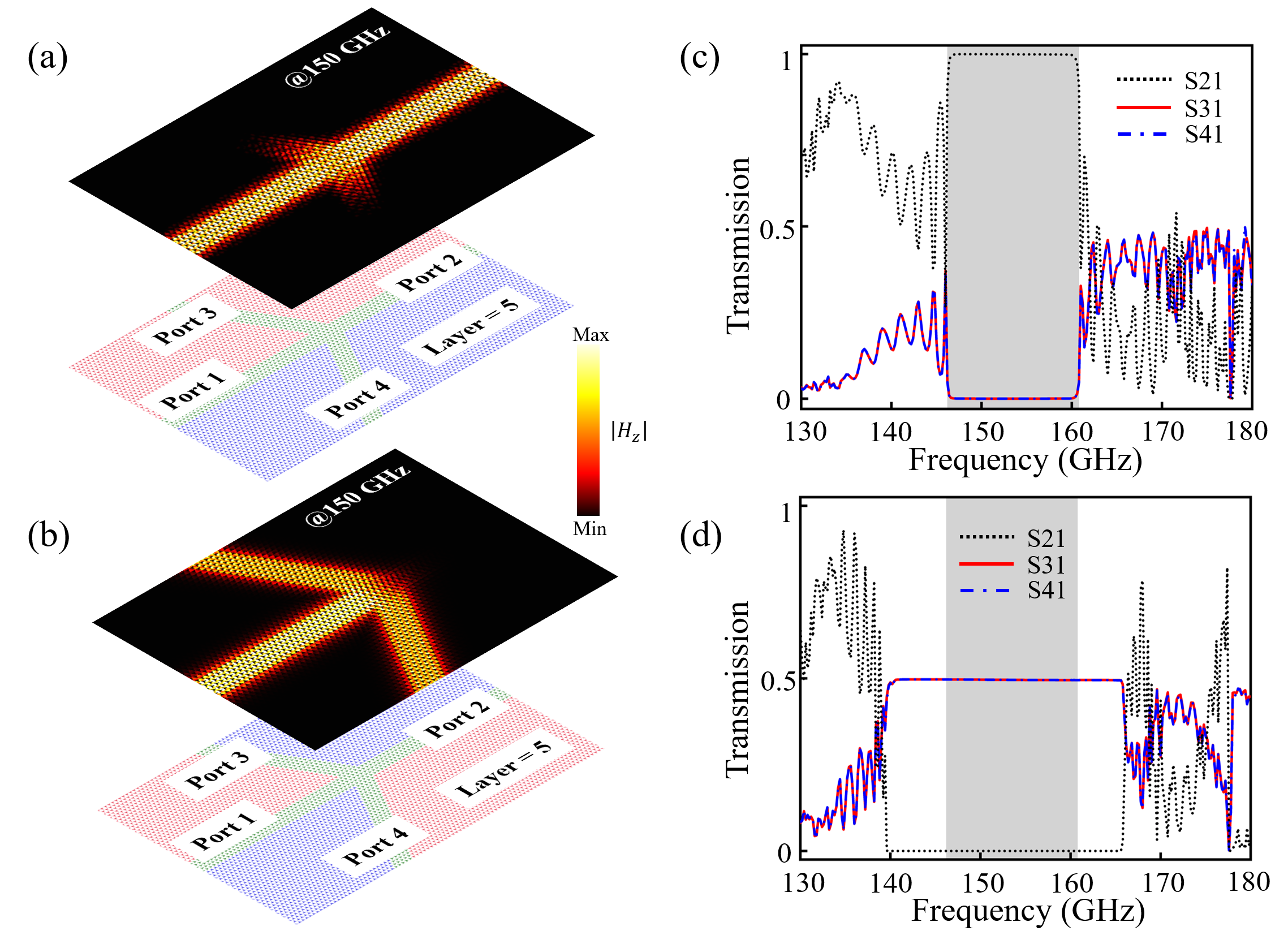


**Fig. S4.** Splitting effect in TVLWs. Upper panels: simulated $\left| H_{z} \right|$ distributions at the frequency of 150 GHz. Lower panels: schematics of TVLWs with $x=5$. (a) AACC, (b) ACCA. Transmission of two valley-locked topological channel. (c) AACC, (d) ACCA. The black, red, and blue lines represent transmission of ports 2-4, respectively. The grey region is the high-efficiency transmission range.

1. The band diagrams of TVLWs with 3 impurities

We further introduce 3 C_4_ impurities to distinguish the specific impacts in TVLWs, which will be utilized in coding channel application. It can be seen that in Fig. S5(a), the impurity modes will add to 3 because of the two more impurities in the super-cell unit. The eigen field distributions at points ‘Ⅰ’, ‘Ⅱ’, ‘Ⅲ’, ‘Ⅳ’, and ‘Ⅴ’ are depicted in Fig. S5(c), which exhibit the different guided mode characteristics. The energy is also confined in the domain B even with 3 ‘ON’ impurities at the TGMs. The energy of impurity modes at three red lines is confined at the positions of impurities, which is resemble as Fig. 3(c). When 3 ‘OFF’ impurities are placed into the super-cell structure, the branches degeneracy points dramatically split (see Fig. S5(b)).


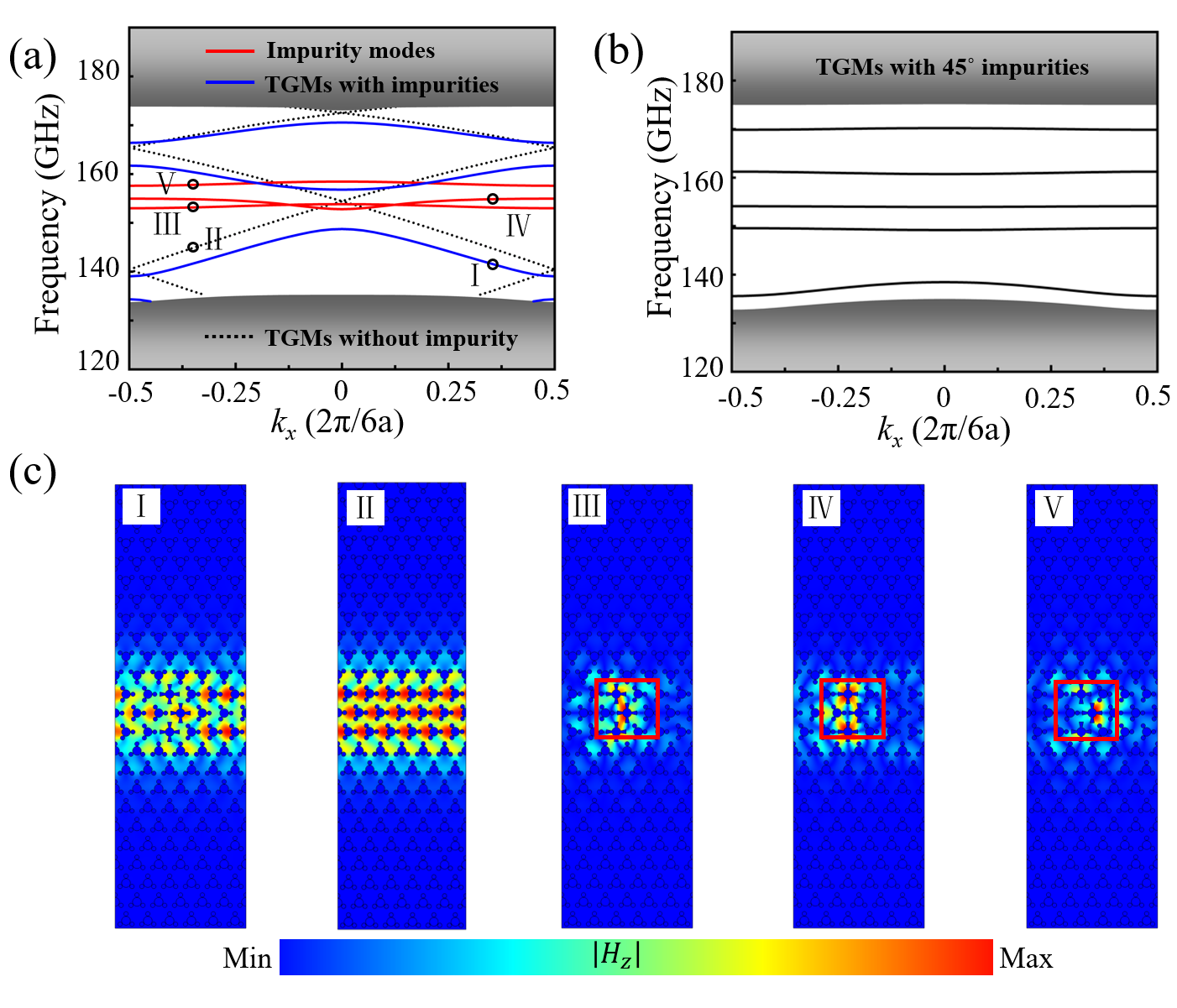


**Fig. S5.** Band diagrams of TVLWs ($A\left| B_{3} \right|C$) with 3 C4 impurities. (a) The band diagrams of super-cell structure without impurity and with 3 ‘ON’ impurities. (b) The band diagrams of super-cell structure with 3 ‘OFF’ impurities. (c) Simulated $\left| H_{z} \right|$ distributions of the super-cell structure at points ‘Ⅰ’, ‘Ⅱ’, ‘Ⅲ’, ‘Ⅳ’, and ‘Ⅴ’ of (a).

1. Forked splitter channel with different input signals

The topologically protected coding channels based on TVLWs with impurity switches have been realized. We first construct a forked splitter channel (see the lower panel in Fig. S6) as the on-chip transmission path based on $A\left| B_{3} \right|C$ TVLWs. When sources are placed at Port 1 to excite as input (red arrow), the energy will propagate along the forked channel and split into Ports 2 and 3 to output (blue arrows), without outputting from Port 4. In addition, when sources are set at Port 2 (Port 3, because of mirror symmetry), the energy will transport to Ports 1, 3 and 4. It can be seen in Fig. S6 (b) that the output energies from different ports are not equal [1]. Furthermore, if we simultaneously put sources at Ports 2 and 3 with different phase differences, the output port will change. Specifically, the phase difference is 0 (π), the corresponding output port is Port 1 (Port 4), as shown in Figs. S6 (c) and (d).


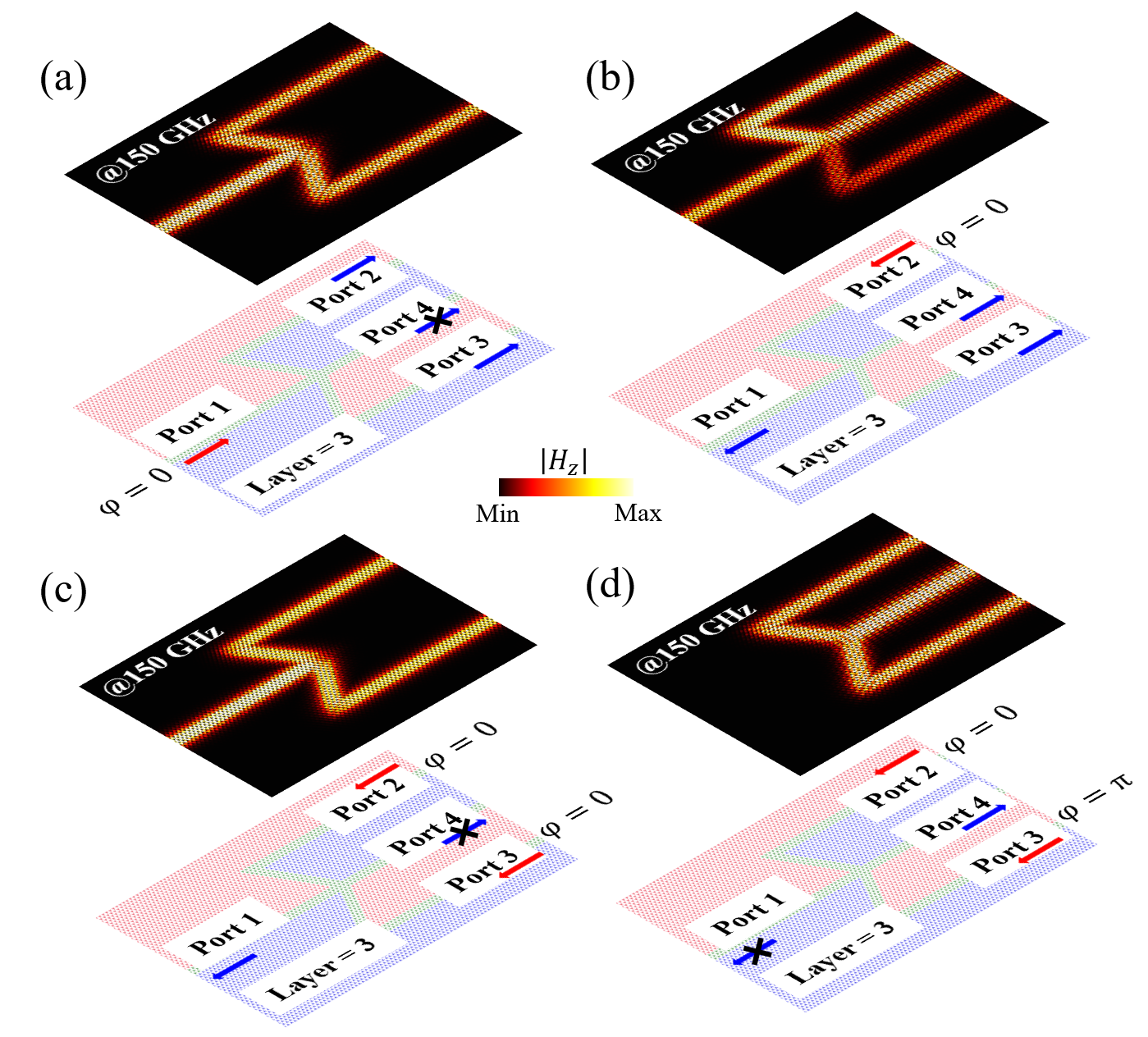


**Fig. S6.** Schematic of forked splitter channel based on TVLWs ($A\left| B_{3} \right|C$). The simulated $\left| H_{z} \right|$ distributions (upper panel) and designed structure (lower panel) of different input ports. The red and blue arrows represent input and output signals, respectively. (a) Port 1. (b) Port 2. (c) Port 2 and Port 3 with phase difference 0. (d) Port 2 and Port 3 with phase difference π.

**Reference**

1. F. Zhang, L. He, H. Zhang, L. Kong, X. Xu, and X. Zhang, "Experimental Realization of Topologically‐Protected All‐Optical Logic Gates Based on Silicon Photonic Crystal Slabs," Laser &amp; Photonics Reviews **17**(8), 2200329 (2023).
